# Physical Methods for Electrical Trap-and-Kill Fly Traps Using Electrified Insulated Conductors

**DOI:** 10.3390/insects13030253

**Published:** 2022-03-03

**Authors:** Yoshinori Matsuda, Teruo Nonomura, Hideyoshi Toyoda

**Affiliations:** 1 Laboratory of Phytoprotection Science and Technology, Faculty of Agriculture, Kindai University, Nara 631-8505, Japan; ymatsuda@nara.kindai.ac.jp; 2 Agricultural Technology and Innovation Research Institute, Kindai University, Nara 631-8505, Japan; 3 Research Association of Electric Field Screen Supporters, Nara 631-8505, Japan; toyoda@nara.kindai.ac.jp

**Keywords:** attractive force, bioelectrical measurement, electric field producer, electricity release, insecticidal function, lethal effect, physical pest control, repulsive force

## Abstract

**Simple Summary:**

Electrostatic insect exclusion is a physical approach to pest control in which an apparatus forming an electric field (EF) is applied to capture pests. Previous studies have clarified the insect-capture mechanisms of such tools and evaluated their practicality. In the present study, we investigated the biological impact of an EF-forming apparatus on adult houseflies captured by the device. We observed the release of substantial levels of negative electricity from the captured flies, detectable as a specific and transient electric current. The electric current was detected at the time of physical attraction, and during subsequent confinement of the fly to the apparatus. The magnitude of electric current from the fly was voltage-dependent, and detrimental effects caused by electricity release became more apparent as the applied voltage increased. Bioelectrical measurements showed that electric current caused acute damage and delayed the death of captured flies. These findings demonstrate the insecticidal function of the insect-capturing apparatus and provide an experimental basis for establishing a tool for trap-and-kill pest management.

**Abstract:**

In the present study, we analyzed negative electricity released from insects captured by an electric field (EF)-producing apparatus. Adult houseflies (*Musca domestica*) were used as the model insect. The EF producer consisted of a negatively charged polyvinyl chloride membrane-insulated iron plate (N-PIP) and a non-insulated grounded iron plate (GIP) paralleled with the N-PIP. An EF was formed in the space between the plates. A housefly placed on the GIP was physically attracted to the N-PIP, and electricity released from the fly was detected as a specific transient electric current at the time of attraction and during subsequent confinement of the fly to the N-PIP. The magnitude of the insect-derived electric current became larger as the voltage applied to the N-PIP increased. We determined the total amount of electric current and confinement time within the apparatus necessary to kill all captured flies. These results demonstrate the insecticidal function and insect-capturing ability of the EF-producing apparatus.

## 1. Introduction

Electrostatic insect exclusion is a physical pest-control approach in which an apparatus forming an electric field (EF) is applied to capture pests. Previous studies have clarified the insect-capture mechanisms of such tools and evaluated their practicality. Some EF-producing pest-capture systems consist of a negatively charged insulated conductor (metal wire or plate) paralleled with a grounded non-insulated conductor; an EF is generated in the space between them [1]. The insects used in such studies have included the whitefly, *Bemisia tabaci* (Gennadius) (Hemiptera: Aleyrodidae) [2,3], vinegar fly, *Drosophila melanogaster* Meigen (Diptera, Drosophilidae) [4,5,6,7], shore fly, *Scatella stagnalis* (Fallén) (Diptera: Ephydridae) [3,7], humpbacked fly, *Megaseria sclaris* (Loew) (Diptera: Phoridae) [7], cigarette beetle, *Lasioderma serricorne* (Fabricius) (Col., Anobiidae) [4,8], Asian tiger mosquito, *Aedes albopictus* (Skuse) (Diptera: Culicidae) [3,9], vegetable leaf miner, *Liriomyza sativae* (Blanchard) (Diptera: Agromyzidae), bathroom fly, *Clogmia albipunctatus* (Williston) (Diptera: Psychodidae), green peach aphid, *Myzus persicae* (Sulzer) (Hemiptera: Aphididae), green rice leafhopper, *Nephotettix cincticeps* (Uhler) (Hemiptera: Deltocephalidae), red flour beetle, *Tribolium castaneum* (Herbst) (Coleoptera: Tenbrionidae), Azuki bean weevil, *Callosobruchus chinensis* Linnaeus (Coleoptera: Bruchidae), oriental termite, *Coptotermes formosanus* Shiraki (Isoptera: Rhinotermitidae), confused flour beetle, *Tribolium confusum* (du Val) (Coleoptera: Tenebrionidae), German cockroach, *Blattella germanica* (Linnaeus) (Orthoptera: Blattellidae), common clothes moth, *Clogmia albipunctatus* Williston (Psychodidae, Diptera), western flower thrip, *Frankliniella occidentalis* (Pergande) (Thysanoptera: Thripidae) [3], and rice weevil *Sitophilus oryzae* (Linnaeus) (Coleoptera: Curculionidae) [3,8]. Electrostatic insect traps were designed to target small, flying insect pests that can pass through conventional insect-proof nets with mesh sizes of 1–1.5 mm. The first designs consisted of an EF screen comprising a layer of insulated conductor wires arrayed in parallel at definite intervals and a parallel grounded metal net [4]. This apparatus was installed on lateral greenhouse windows to prevent pest entry [10,11]. The EF screen technique has been applied in an electrostatic nursery shelter to protect tomato seedlings from whiteflies, leaf miners, aphids, and thrips in an open-window greenhouse environment [12], a portable electrostatic insect sweeper to trap whiteflies colonizing host plants [13], and an electrostatic seedbed cover to capture leaf miners emerging from underground pupae [14]. In this system, a negative voltage generator picks up negative charge from the ground and supplies it to a linked insulated conductor that accumulates negative charge at its outer surface, dielectrically polarizing the insulator cover to generate the negative charge [15]. This negative charge positively polarizes a grounded conductor through electrostatic induction [16]. These opposite charges generates an EF in the space between the opposite poles (i.e., the negatively charged insulated and positively charged grounded conductors).

Charged poles within the EF generate an attractive or repulsive force to other charges in the field [17]; these forces may be involved in insect capture within the apparatus [3,5,6]. The negatively charged insulated conductor pushes free electrons (negative electricity) out of an insect that enters the EF and sends them to the ground via a grounded conductor; such events are detected as a transient electric current from the insect. We hypothesized that the insect is subjected to discharge-mediated positive electrification and then attracted to the negatively charged conductor [1]. The force generated during this process is sufficiently strong to prevent insects from escaping the trap, making the electrostatic insect trap practically applicable for a wide range of insect pests [18].

Despite active research on the insect-capturing mechanism of EF-producing insect pest-control devices and their practical applications in pest control, no studies have reported the biological impact of these devices on insects. Thus, the main objective of the present study was to determine whether electrostatic insect-capturing devices are insecticidal. As the intensity of an EF (i.e., the force required to push negative electricity out of the insect) is determined as a transient electric current from the insect [4,6,10], it is essential to clarify the relationships between the magnitude of the insect-mediated electric current and voltage applied to an insulated conductor, and between the current magnitude and captured insect survival. Clarifying the length of time required for captured insects to be killed is vital to understand the insecticidal function of the electrostatic insect-capturing apparatus. In this study, we used the adult housefly, *Musca domestica* (Linnaeus) (Diptera: Muscidae), as a model insect because larger insects produce larger amounts of transient electric current within the EF [3]. Moreover, the housefly is considerably larger than many insect species captured by the present apparatus. The EF was constructed using a pair of insulated and non-insulated iron plates of the same size, to maintain an identical pole distance between the charged, insulated and grounded plates [19]. Using the obtained results, we then described the insecticidal function of the electrostatic insect-capturing apparatus to provide an experimental basis for a trap-and-kill method for a broad range of insect pest species.

## 2. Materials and Methods

### 2.1. Insect

**Figure 1:**
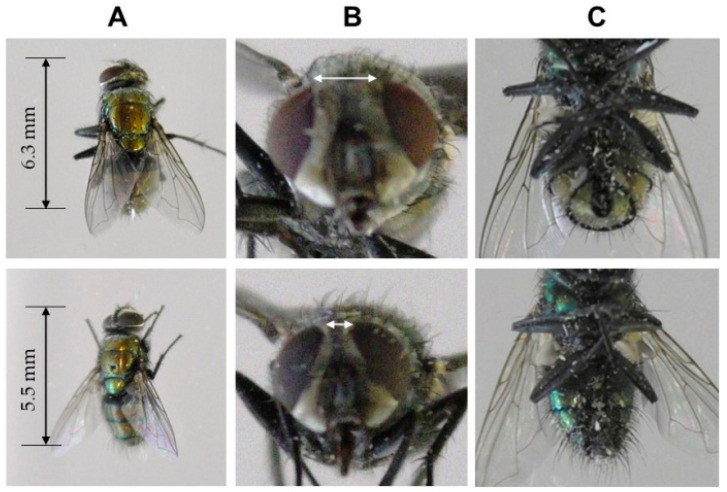
Sexual dimorphism in the external morphology of female (**upper**) and male (**lower row**) adult houseflies (*Musca domestica*). (**A**) Female and male body sizes (dorsal view). (**B**) Female and male eye spacing (front view). (**C**) Female with post-abdomen ovipositor and male with post-abdomen genital appendages (ventral view).

Adult houseflies (*M. domestica*) were purchased from Sumika Technoservice (Hyogo, Japan) and reared on a certified diet (MF; Oriental Yeast Co., Ltd., Tokyo, Japan) [20] in a closed 30-mL transparent acrylic vessel. Insect rearing was conducted in a growth chamber (25 ± 0.5 °C, 12 h photoperiod, 4000 lux) from the egg to adult stages. Pupae found on the medium were individually transferred onto fresh medium in a 20-mL vial for isolation, and the vial mouth was covered with gauze. The sex of adult flies emerging from the pupal stage was determined based on the sexual dimorphism of the external morphology of *M. domestica* [21], as shown in Figure 1. The average survival rates of adult male and female houseflies were 31.5 ± 0.8 and 32.3 ± 0.6 days after eclosion, respectively. In the present study, male and female adult houseflies of various ages (7–21 days after eclosion) were used for the evaluation of fly capturing efficiency and electric-current generation (by the fly). Fly mortality was also assessed. In these experiments, 20 flies were used for each sex, age, and applied voltage.

### 2.2. EF Production

**Figure 2:**
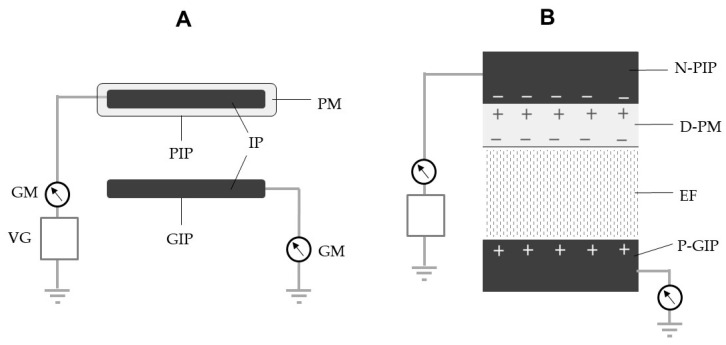
Schematic representation of (**A**) an electric field producer (EFP) and (**B**) dielectric polarization of a polyvinyl chloride (PVC) membrane used to insulate an iron plate, followed by electrostatic induction of a grounded iron plate paralleled with an insulated iron plate. D-PM, dielectrically polarized PVC membrane; EF, electric field; GIP, grounded iron plate; GM, galvanometer; IP, iron plate; N-PIP, negatively charged iron insulated iron plate; P-GIP, positively polarized grounded plate; PIP, PVC-insulated iron plate (charged conductor); PM, PVC membrane coating (insulator); VG, voltage generator.

The structure of the EF producer (EFP) is shown in Figure 2A. Two identical iron plates (2 × 10 cm^2^, 2 mm thickness) were used to construct the EFP; one was coated with a soft polyvinyl chloride (PVC) membrane (1 mm thickness; 10^9^ Ω cm) (Sonoda Seisakusho, Osaka, Japan) for insulation and linked to a negative voltage generator (Max Electronics, Tokyo, Japan), while the other was non-insulated and linked to a grounded line. The plates were arranged in parallel at a distance of 10 mm. A transformer and Cockcroft circuit [22] were integrated so as to enhance the initial voltage (12 V) of the voltage generator to achieve the desired voltages (−1 to −20 kV). With this enhanced voltage, the generator is able to pick up negative electricity from the ground and supply it to a PVC-insulated iron plate (PIP) [23]. Negative electricity accumulates on the surface of the iron plate and polarizes the conductor-side surface (positive) and outer surface of the insulator coating (negative). Eventually, the negative surface charge polarizes the non-insulated grounded iron plate (GIP), so that it is positively charged through electrostatic induction [16]. The opposite charges on the PIP and GIP generate an EF in the space between them (Figure 2B).

We applied voltages between −1 and −15 kV to generate an electric current via the N-PIP to evaluate insect capture, and voltages between −8 and −15 kV to evaluate current generation by the fly and fly mortality. All experiments were conducted in a room controlled at 25 ± 0.5 °C and 60% relative humidity.

### 2.3. Interception of the Flow of Electric Current Generated by Silent Discharge of the N-PIP

**Figure 3:**
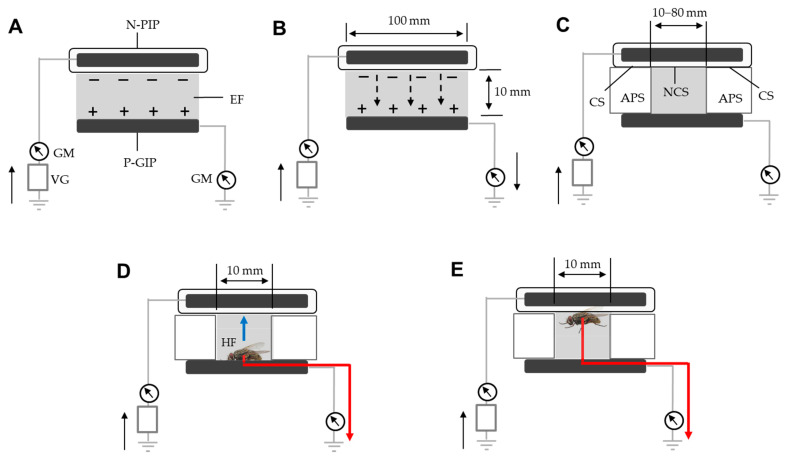
Electric current generation by instrument- and insect-derived discharge in an EFP negatively charged by different applied voltages. (**A**) No discharge occurs from the negatively charged iron plate in the lower voltage range (−1 to −7.9 kV), where an insulating PVC membrane prevents the release of negative electricity from the charged iron plate. (**B**) Silent discharge occurs at >−8.0 kV, and the amount of charge released increases in direct proportion to the increase in applied voltage. This discharge was recorded as a stable electric current to the ground by a galvanometer integrated into the ground line of the grounded iron plate. (**C**) Acrylic plate spacer (APS) insulators inserted between the N-PIP and P-GIP to intercept electricity released from the closed surface of the N-PIP at higher voltages (−8 to −15 kV). (**D**) Housefly (HF)-derived discharge occurs when the fly is attracted to the N-PIP (blue arrow). This discharge is recorded as a specific transient electric current. (**E**) Subsequent discharge from the attracted fly. Solid black arrow indicates the direction of electricity movement from ground to ground. Dotted black arrow indicates electricity movement through silent discharge. Red arrow indicates insect-derived electric current. APS, acrylic plate spacer; CS, closed surface of the N-PIP; EF, electric field; GM, galvanometer; NCS, non-closed surface of the N-PIP; N-PIP, negatively charged PVC-insulated iron plate; P-GIP, positively charged grounded iron plate; VG, voltage generator.

Within a certain range of the applied voltage, the insulation of the charged iron plate prevents the transfer of the negative charge from the N-PIP to the opposite pole (GIP), i.e., discharge from the PIP to the ground via the GIP (Figure 3A). However, if the applied voltage exceeds the limit, the negative charge on the iron plate passes through the insulation toward the ground via the GIP (discharge of the PIP). This movement of electricity was detected as an electric current using two galvanometers (Sanwa, Tokyo, Japan) integrated into the grounded lines of the voltage generator and GIP (Figure 3B). This type of discharge has been described as the silent discharge of the N-PIP [24].

First, we examined the range of voltages that caused silent discharge by gradually increasing the voltage applied to the PIP. The resulting electric current profiles were recorded using a current detector (detection limit, 0.01 µA) integrated into the galvanometer. We also determined the voltage range that caused arc (spark) discharge by further increasing the applied voltage.

Next, we inserted two identical acrylic plates (10^12^ Ω cm) into the space between the PIP and GIP to determine the degree to which electricity released from the N-PIP was intercepted (Figure 3C). For this purpose, we used an acrylic plate with a thickness of 10 mm, width of 20 mm, and length of 60–95 mm. Finally, we prepared EFPs, each with a PIP with a non-closed surface of varying length (range, 10–80 mm) at the central region (Figure 3C), and measured the magnitude of electric current over a range of voltages (−8 to −15 kV) causing silent discharge in the EFP with the non-closed PIP.

### 2.4. Attraction of Flies to the N-PIP of the EFP

In this study, we used the EFP shown in Figure 3D, for which the central 200-mm^2^ area of the PIP was not closed. The EFP was negatively charged with different voltages (−1 to −15 kV), and single male and female houseflies of various ages (7, 14, and 21 days after eclosion) were then transferred onto the P-GIP to determine the voltage range within which the flies were physically attracted to the N-PIP. Flies that were attracted to the N-PIP were continuously observed for 1 h to determine whether they could escape the N-PIP. When flies were confined to the N-PIP during the observation period, capture was considered successful. Thus, we precisely determined the voltage range at which 100% of male and female houseflies of various ages were captured.

### 2.5. Measurement of Electric Current from the Attracted Houseflies

**Figure 4:**
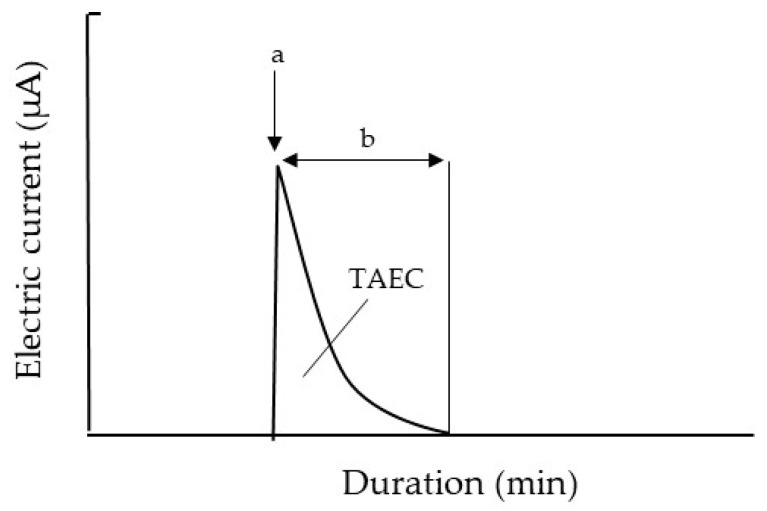
Typical profile of electric current generated by an adult housefly placed on the GIP of the negatively charged EFP. (a) Electric current produced by the fly at the time of attraction to the N-PIP. (b) The electric current produced during subsequent confinement to the N-PIP. The total amount of electricity released from the fly was calculated as the total amount of electric current (TAEC) (µA min) generated by the fly, according to the area bounded by the x-axis and the plotted curve of the generated current.

Using the same set of houseflies described in Section 2.4, we transferred each fly onto the GIP of the EFP (Figure 3D), which was negatively charged at different voltages (−8 to −15 kV), and recorded the occurrence of the electric current from the captured insect using the current recorder described above. Figure 4 shows a typical electric current profile recorded from a fly. The moment the fly was placed on the GIP, the electric current increased rapidly and then declined gradually. The first current peak represented the initial release of electricity from the fly body, electrifying it positively; this peak was detected when the fly was attracted to the N-PIP. The subsequent current was derived from the fly captured by the N-PIP. In this experiment, we determined the electric current at the first peak, and the duration of the subsequent current for each voltage setting.

### 2.6. Ascertainment of Fly Mortality following Capture by the N-PIP

In this experiment, we used the modified EFP (Figure 3C) charged with different voltages (−8 to −15 kV), and continuously observed a housefly captured with the N-PIP to determine its time of death under each voltage setting. The death of the captured fly was ascertained by gently touching its body with an acrylic rod (insulator). Living flies produced convulsive limb movements in response to this stimulus, whereas dead flies remained motionless. Mortality was further confirmed by releasing the fly from the force of the N-PIP. We also examined the correlation between the total amount of negative electricity released from the fly body and the time until death. The total amount of electricity released was determined as the total amount of electric current (TAEC) released from the fly, calculated according to the area bounded by the x-axis and a curve that was plotted using the trapezoid rule (Figure 4) [25]. In the final experiment, we examined the survival of houseflies released from the N-PIP at various intervals after current-generation from the captured fly had ceased. The 7-day-old male and female houseflies were attracted to the N-PIP charged at −8 kV, and then released from the N-PIP by stopping the voltage application at various intervals (range: 1–7 h) after insect current-generation had ceased. Fly mortality was evaluated at 5 h after release from the N-PIP.

### 2.7. Statistical Analysis

In all experiments, 20 insects were used for each sex, age, and applied voltage. All experiments were repeated five times, and data are presented as means ± standard deviation. Tukey’s test and linear regression analysis were performed using EZR software v1.54 (Jichi Medical University, Saitama, Japan) to identify significant differences among conditions and any correlations among the factors.

## 3. Results and Discussion

### 3.1. Interception of Electricity Released from the N-PIP by the Insulator Spacer

**Figure 5:**
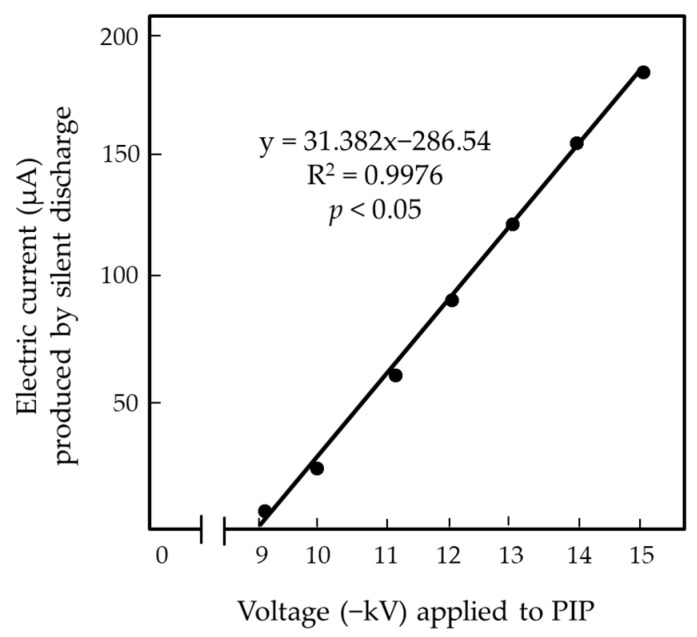
Linear correlation between the voltage applied to the PIP of the EFP and the magnitude of electric current generated by silent discharge from the negatively charged PIP.

The primary objective of the present study was to suppress the flow of the electric current generated by the mechanical discharge of the N-PIP, which involved both silent and arc discharges. The amount of negative charge that accumulated on the surface of the PIP increased in direct proportion to the applied voltage. The voltage generator used in this study supplied voltages ranging from −1 and −20 kV. However, at >−15.1 kV, the insulator membrane that covered the PIP to generate arc discharge from the N-PIP broke down. Ultimately, we decided not to use this voltage range due to direct exposure of the insect to the arc discharge. In fact, flies exposed to arc discharge tend to be expelled from the EF due to its strong impact [19,26]. At <−8.5 kV, the PVC membrane was sufficiently insulative to suppress the release of negative charge from the N-PIP; therefore, this voltage range was used in the present study (Figure 3A). At voltages between −8.5 and −15 kV, the PVC membrane was less insulative, such that negative charge on the iron plate moved to ground due to the silent discharge of the N-PIP (Figure 3B). This electricity movement was detected as an electric current by a galvanometer integrated into the grounded line of the GIP. Silent discharge continued stably while the voltage was applied, and there was a linear correlation between the magnitude of the electric current and the applied voltage (Figure 5).

Silent discharge of the N-PIP can occur along the entire surface facing the P-GIP. To intercept the electricity released from the N-PIP, we partially closed the surface of the N-PIP using an acrylic plate (Figure 3C). High resistivity (10^12^ Ω cm) of the acrylic plate indicated high insulative properties [27], which suppressed the movement of electricity from the closed surface. We examined the change in current magnitude of the silent discharge of the N-PIP by gradually narrowing the non-closed surface (Table 1). As expected, the current magnitude became smaller as the non-closed surface of the N-PIP decreased, finally becoming undetectable at an area of 200 mm^2^, which was used for subsequent insect capture and insect-derived current flow experiments.

### 3.2. Attraction of Houseflies to the N-PIP

One of the most important events for pest control by an EFP is the attraction of the insect to the negatively charged insulated conductor within the EF [1]. In the present EFP, the attraction of a housefly to the N-PIP was detected in the range from −5.5 to −15 kV. However, at <−7.6 kV, the attracted flies were able to escape from the N-PIP within a short time. Table 2 lists the time required for houseflies to escape following their attraction to the N-PIP of the negatively charged EFP at different voltages. In this experiment, male and female adult houseflies of different ages were used to clarify the difference in insect-capturing efficiency across different voltage conditions. In male flies, attraction to the N-PIP was first detected at −5.5 kV, whereas a larger voltage (−6 kV) was required to attract female flies, which are larger than males. In both cases, the attracted flies took longer to escape at a higher applied voltage. The force of the N-PIP impeding the movement of the fly became larger as the applied voltage increased. As the females escaped sooner than the males at all applied voltages, females appear to exert a larger force opposing that of the N-PIP than males. Video S1A shows the movement of a 7-day-old female fly attracted to the N-PIP at −6 kV; the fly struggled and then escaped the trap. All flies that escaped from the trap were confirmed to survive the following 5-day observation period, regardless of sex, age, or applied voltage. At >−8 kV, the N-PIP exerted a stronger force to confine the fly, such that no flies escaped the EFP, even after 1 day, had elapsed. Video S1B shows fly capture at −8 kV.

### 3.3. Electric Current Generation by a Housefly Attracted to the N-PIP 

Flies placed in the EF were exposed to a repulsive force from the N-PIP, such that free electrons in the insect body were pushed out of the fly toward the ground via the GIP. This electricity movement was recorded as a transient electric current from the insect [4,6,10]. Eventually, the insect was positively electrified and attracted to the N-PIP. The outer protective cuticle of many invertebrates can be efficiently electrified due to its conductive nature [19,28,29,30,31,32]. Therefore, we explored insect conductivity in terms of electricity release from the insect body within the EF.

**Figure 6:**
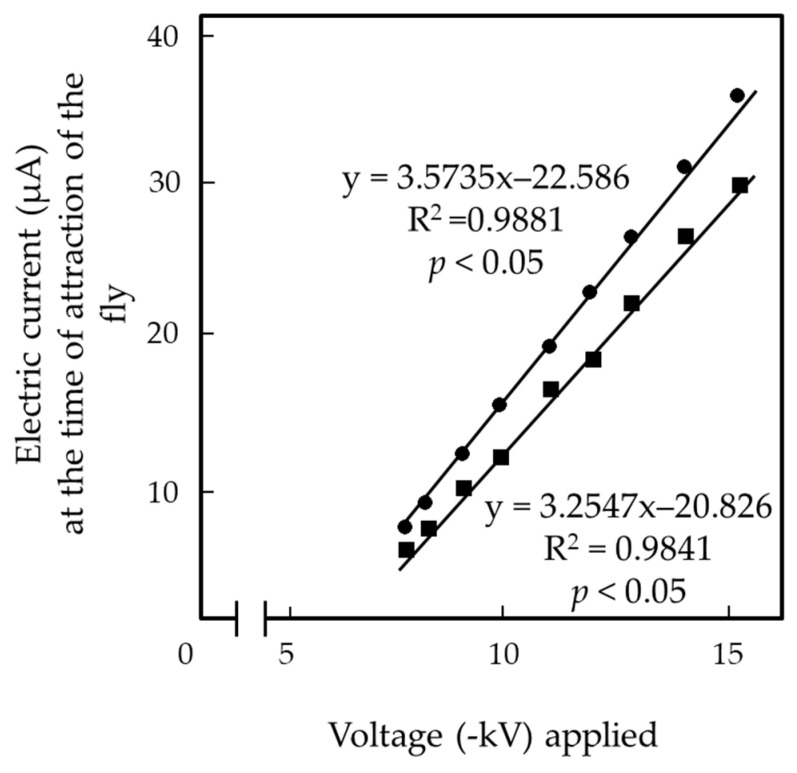
Linear correlation between the voltage applied to the PIP of the EFP and the electric current generated by 7-day-old male and female houseflies upon attraction to the N-PIP.

We measured the electric current from houseflies using a modified EFP (Figure 3D) charged with −8, −10, −12, and −15 kV, and the same set of the houseflies used in the previous experiment. Table 3 shows the electric current at the first peak (Figure 4), which represents the initial release of the electricity from the insect body used to electrify it positively. This peak was detected when the fly was attracted to the N-PIP. Table 3 also shows the subsequent electric current from the fly captured with the N-PIP (Figure 4). The height of the first peak (i.e., current magnitude) and duration of continuous current increased as the applied voltage increased. Figure 6 shows the linear relationship between the applied voltage and current magnitude generated by a single fly at the time of attraction over the entire voltage range for insect attraction. Females showed significantly larger current magnitude and a longer current generation duration than males; however, neither current magnitude nor duration differed significantly among flies of different ages within each sex. These results strongly support our hypothesis that houseflies are positively charged due to the release of electricity from the insect body that occurred immediately after its transfer to the EF. Continuously depriving the fly of its electricity appeared to strengthen the force constraining the fly to the N-PIP.

### 3.4. Release of Negative Electricity from the Insect Body Was the Primary Cause of Death of Captured Houseflies

**Figure 7:**
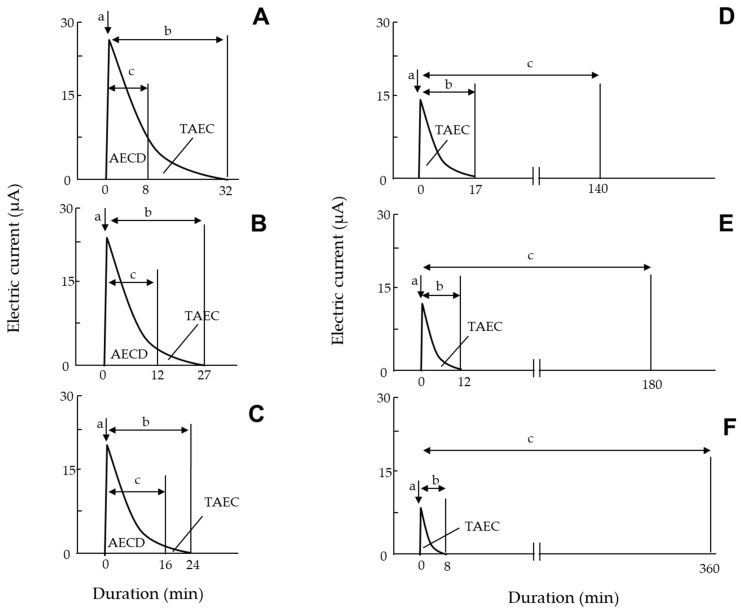
Typical profiles of electric current generated by an adult housefly placed on the GIP of an EFP negatively charged with (**A**) −15, (**B**) −14.5, (**C**) −14, (**D**) −12, (**E**) −10, and (**F**) −8 kV. Arrows a and b indicate the electric current produced by the fly upon attraction to the N-PIP and the subsequent electric current generated by the captured fly, respectively. The total amount of electricity released from the captured fly was determined as the total amount of electric current (TAEC; µA min) generated by the fly, and calculated as the area bounded by the x-axis and the profile curve of current generation. Arrow c indicates the time until death of the captured fly. The total amount of electric current (AECD) until fly death was also calculated.

Our findings indicated that insect-derived electric-current generation caused both an attraction and confinement of the fly to the N-PIP within an applied voltage range of −8 to −15 kV. Houseflies captured by the N-PIP remained positively electrified until the voltage supply to the N-PIP ended. We evaluated the effect of this electrification on the fly in terms of the total amount of negative electricity released therefrom, calculated as the product of the magnitude of electric current and the duration of current generation, which was equivalent to the area bounded by the x-axis and the current generation curve (Figure 4). Figure 7 shows typical profiles of the electric current generated by a housefly captured by the N-PIP over an applied voltage range of −8 to −15 kV, including the time to the end of electric-current generation by the fly and death of the fly. At higher applied voltages (−14, −14.5, and −15 kV), fly death occurred later as the applied voltage decreased, such that flies were killed before the electric current ceased (Figure 7A–C). At lower applied voltages (−8, −10, and −12 kV), flies survived even after current generation from the fly had ended (Figure 7D–F). However, all tested flies were ultimately killed by the apparatus, despite variations in survival time among the applied voltages.

Table S1 lists the TAEC generated by the captured flies and the amount of electric current generated until death (AECD), as well as the time until current generation ended (Figure 7). At all applied voltages, TAEC was significantly larger in females than males, and time until death was significantly longer in females than males, indicating that female flies were more tolerant to electricity-release-mediated damage than males. However, there was no significant difference in TAEC or time until death among flies of different ages within the same sex. Notably, fly mortality and the AECD were closely related; the AECD of flies captured with the N-PIP at −14, −14.5, and −15 kV was very similar (Table S1). Because the flies died when the electricity release reached the TEAC, we concluded that the lethal amount of electricity released from the fly body was approximately 120 µA min for adult males and females. However, at lower voltages (−8 to −12 kV), the electricity release ceased before the AECD reached a lethal amount of electric current. Nevertheless, the electricity release was detrimental within this range of the applied voltage. In fact, all flies were dead during capture by the N-PIP. In both male and female flies, the time to death decreased as the applied voltage increased (Figure 7D–F; Table S1).

**Figure 8:**
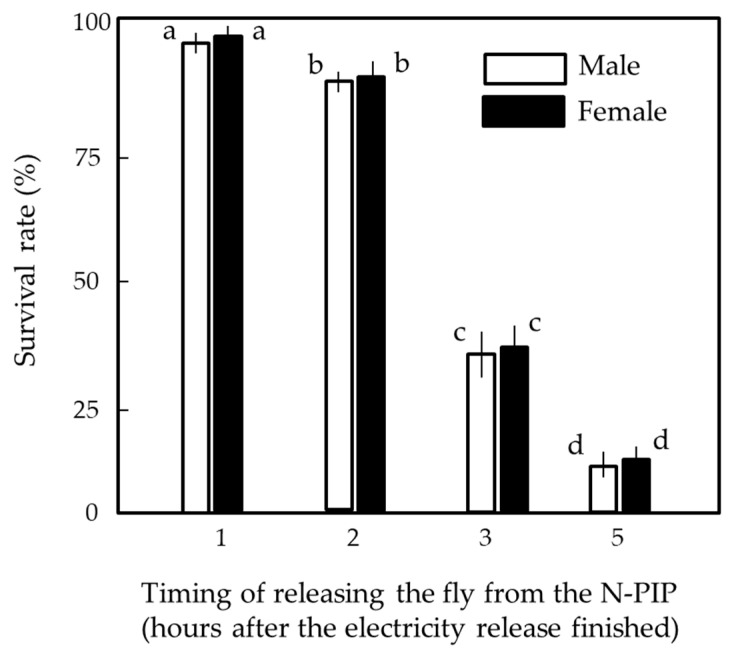
Relationship between the time of release of 7-day-old female houseflies from the N-PIP charged at −8 kV and survival. Fly mortality was determined at 5 days after release from the N-PIP. We used 20 insects for each sex and hour, and means ± standard deviation were calculated from five experimental replicates. Letters (a–d) in each vertical column indicate significant differences (*p* < 0.05) according to Tukey’s test.

One fly was released from the N-PIP before it was killed. In the final experiment, after electric-current generation by the fly had ceased and before its death, we switched off the voltage generator to determine whether it was alive or dead. Switching off the voltage generator implies the removal of a repulsive force used to push negative electricity out of the insect body. The positively polarized body likely attracted free electrons from the air [33], restoring the fly to its original state via neutralization. Figure 8 shows the survival rates of 7-day-old male and female houseflies released from the N-PIP (charged at −8 kV) at different times after current generation by the flies had ended. The detrimental effects persisted even after the force had been removed, and some flies died within 5 h of release. The fly mortality rate increased as the release time was delayed. There was no significant difference in the survival rate between male and female flies. Thus, it appears that the lethal effect of the apparatus persisted even if flies were restored from their positively electrified state.

In the present study, we demonstrated that houseflies in an EF produced by the N-PIP and P-GIP of the EFP were captured by the N-PIP. The present bioelectrical method was useful to confirm the release of negative electricity from the fly, in the form of the electric-current flow from the insect body. This electric-current generation was successfully shown to correspond to the attraction and subsequent confinement of a fly to the N-PIP. These results strongly support our hypothesis that the negative charge of the PIP generates a repulsive force that pushes negative electricity (free electrons) out of the insect body; insects that lost their negative electricity became positively charged and were attracted to the N-PIP [1]. The main finding of this study was a causal relationship between electricity release from the captured flies and death during capture or after release from the trap. Our previous pest-control studies aimed to enhance the insect-trapping ability of EFP apparatuses [18]. Such apparatuses are applicable to many insect pest species [3]; therefore, the bifunctional ability of the EFP apparatus tested in the present study will contribute to the further development of these trap-and-kill tools for use against more insect pest species.

The next stage in the design of the EFP apparatus is to develop a new contraceptive technique for insect pests. In a preliminary experiment, we determined that some flies laid no eggs during the entire experimental period following intermittent exposure to the attractive force of the N-PIP at an applied voltage of approximately −6 kV. In a future study, we will further analyze the biological impact of the attractive force of the apparatus on the fecundity of houseflies placed within an EF. The findings of this study may facilitate the application of electrostatic-based pest-control methods to many insect species.

## 4. Conclusions

In the present study, an EF was formed in the space between a negatively charged PIP and GIP within an EF producer. The release of negative electricity from houseflies was detected as they were first attracted, and subsequently confined, to the negatively charged iron plate. This electricity release was detected as the electric current from the fly to the ground via the GIP. The release of electricity from the insect body was dependent upon the applied voltage, and its detrimental effect became increasingly noticeable as the voltage increased. The present study clarified the lethality of the electric current to the housefly, regardless of sex or age.

## Figures and Tables

**Table 1:** Interception of current flow generated through silent discharge of a negatively charged polyvinyl chloride (PVC)-insulated iron plate (N-PIP) of an electric field producer (EFP), achieved by closing the surface of the N-PIP with acrylic plates at different degrees.

Area (mm^2^) of Non-Closed Surface of the N-PIP	Magnitude (µA) of Electric Current Generated by Silent Discharge of the N-PIP							
	8	9	10	11	12	13	14	15 ^a^
2000 (no spacer, control)	0 ^b^	1.6 ± 0.5	22.7 ± 2.7	56.2 ± 1.3	89.5 ± 1.2	121.1 ± 1.1	151.7 ± 5.8	186.6 ± 2.3
1600	0	1.2 ± 0.3	18.1 ± 1.2	45.1 ± 0.6	72.2 ± 0.8	97.7 ± 1.0	122.2 ± 4.4	150.1 ± 1.3
1200	0	1.1 ± 0.2	14.1 ± 1.3	34.5 ± 1.1	54.7 ± 1.3	72.9 ± 0.9	93.3 ± 3.4	113.0 ± 1.3
1000	0	0.5 ± 0.1	11.8 ± 1.0	28.8 ± 0.6	44.9 ± 0.8	61.3 ± 0.8	78.1 ± 1.8	94.0 ± 1.1
800	0	0	9.4 ± 0.9	22.9 ± 0.6	36.2 ± 0.8	48.5 ± 0.8	62.1 ± 1.3	75.4 ± 0.7
600	0	0	1.9 ± 0.9	16.8 ± 0.7	26.4 ± 0.8	36.4 ± 0.4	45.5 ± 1.6	56.5 ± 0.7
400	0	0	0	0	4.6 ± 0.5	17.4 ± 0.7	24.4 ± 0.4	30.1 ± 1.2
200	0	0	0	0	0	0	0	0

^a^ Negative voltage (-kV) applied to the N-PIP; ^b^ The current recorder displayed zero for magnitudes below the detectable limit (0.01 µA).

**Table 2:** Time (s) required for male and female adult houseflies captured with the negatively charged PIP of the EFP at different voltages to escape from the PIP.

Sex	Age ^1^	Voltage (-kV) Applied to the PIP									
		5	5.5	6	6.5	7	7.5	8	10	12	15
Male	7	n.a. ^2^	2.6 ± 0.7 a	4.1 ± 0.3 a	5.2 ± 0.4 a	6.9 ± 0.3 a	7.8 ± 0.6 a	n.a.e. ^3^	n.a.e.	n.a.e.	n.a.e.
	14	n.a.	2.7 ± 0.8 a	4.2 ± 0.4 a	5.3 ± 0.5 a	6.8 ± 0.4 a	7.7 ± 0.5 a	n.a.e.	n.a.e.	n.a.e.	n.a.e.
	21	n.a.	2.4 ± 0.7 a	4.5 ± 0.5 a	5.4 ± 0.5 a	6.9 ± 0.6 a	7.9 ± 0.6 a	n.a.e	n.a.e.	n.a.e.	n.a.e.
Female	7	n.a.	n.a.	3.1 ± 0.3 b	3.7 ± 0.5 b	5.3 ± 0.5 b	5.9 ± 0.3 b	n.a.e.	n.a.e.	n.a.e.	n.a.e.
	14	n.a.	n.a.	3.2 ± 0.4 b	3.6 ± 0.5 b	5.4 ± 0.5 b	5.9 ± 0.6 b	n.a.e.	n.a.e.	n.a.e.	n.a.e.
	21	n.a.	n.a.	3.3 ± 0.5 b	3.5 ± 0.5 b	5.2 ± 0.4 b	5.9 ± 0.6 b	n.a.e.	n.a.e.	n.a.e.	n.a.e.

^1^ Days after eclosion; ^2^ Flies were not attracted; ^3^ Flies were attracted to the PIP but not allowed to escape from the trap. We used 20 insects for each sex, age, and applied voltage. Means ± standard deviation were calculated from five experimental replicates. Different letters (a, b) within each column indicate significant differences (*p* < 0.05) according to Tukey’s test.

**Table 3:** Magnitude of electric current generated upon the attraction of male and female adult houseflies to the N-PIP of the EFP, and duration of the subsequent current generation by the flies captured by the N-PIP.

Sex	Age ^1^	Magnitude (µA) of Electric Current				Duration (min) of Electric Current Generation			
		8	10	12	15 ^2^	8	10	12	15 ^2^
Male	7	4.9 ± 0.4 a	9.2 ± 0.2 a	13.4 ± 0.3 a	27.6 ± 1.5 a	6.4 ± 0.3 a	10.6 ± 0.4 a	15.3 ± 0.4 a	28.3 ± 0.5 a
	14	4.8 ± 0.1 a	9.3 ± 0.2 a	13.9 ± 0.2 a	27.8 ± 1.2 a	6.1 ± 0.3 a	10.5 ± 0.3 a	15.6 ± 0.3 a	28.7 ± 0.6 a
	21	4.7 ± 0.3 a	9.1 ± 0.3 a	13.8 ± 0.2 a	27.0 ± 1.6 a	6.2 ± 0.2 a	10.7 ± 0.5 a	15.9 ± 0.5 a	28.2 ± 0.4 a
Female	7	6.3 ± 0.2 b	11.8 ± 0.2 b	16.8 ± 0.5 b	35.0 ± 1.3 b	7.8 ± 0.2 b	12.5 ± 0.1 b	17.7 ± 0.3 b	32.1 ± 0.4 b
	14	6.3 ± 0.1 b	11.9 ± 0.3 b	16.6 ± 0.2 b	35.8 ± 1.2 b	7.9 ± 0.5 b	12.2 ± 0.3 b	17.5 ± 0.4 b	32.0 ± 0.5 b
	21	6.2 ± 0.3 b	11.7 ± 0.2 b	16.5 ± 0.4 b	35.1 ± 1.4 b	8.1 ± 0.6 b	12.1 ± 0.2 b	17.8 ± 0.2 b	32.3 ± 0.6 b

^1^ Days after ecolosion; ^2^ Negative voltages applied to the PIP. We used 20 insects for each sex, age, and applied voltage. Means ± standard deviation were calculated from five experimental replicates. Different letters (a, b) within a column indicate significant differences (*p* < 0.05) according to Tukey’s test.

## Supplementary Materials

The following are available online at https://www.mdpi.com/article/10.3390/insects13030253/s1, Table S1: Relationship between electric current generation by houseflies confined to a polyvinyl chloride (PVC)-insulated iron plate (N-PIP) of an electric field producer (EFP) negatively charged at different voltages and time of housefly death, Video S1: (A) Incomplete and (B) complete capture of 7-day-old female flies with a polyvinyl chloride (PVC)-insulated iron plate (PIP) of an electric field producer (EFP) charged at −6 and −8 kV, respectively.
