# Supplementary material for: Physical Methods for Electrical Trap-and-Kill Fly Traps Using Electrified Insulated Conductors
Source: Insects. 2022 Mar 3;13(3):253. doi: 10.3390/insects13030253 (PMC8950617; doi:10.3390/insects13030253)
Supplement: Supplementary file 1 [file insects-13-00253-s001.zip › Table S1.pdf]

Table S1. Relationship between electric current generation by houseflies confined to a polyvinyl chloride (PVC)-insulated iron plate (N-PIP) of an electric field producer (EFP) negatively charged at different voltages and time of housefly death.

Voltage (-kV) applied to N-PIP	Sex	Age [1]	Total amounts (μA·min) of electric current to		Time length (min) to	
			termination of current generation (TAEC)[2]	death (AECD)[2]	termination of current generation	death
8	Male	7	14.7 ± 0.3 a	14.7 ± 0.3 a	6.1 ± 0.3 a	321.0 ± 2.8 a
		14	15.1 ± 0.5 a	15.1 ± 0.5 a	6.2 ± 0.2 a	329.1 ± 3.0 a
		21	15.4 ± 0.4 a	15.4 ± 0.4 a	6.1 ± 0.3 a	327.0 ± 2.4 a
	Female	7	17.5 ± 0.3 b	17.5 ± 0.3 b	7.8 ± 0.1 b	354.7 ± 3.1 b
		14	17.2 ± 0.4 b	17.2 ± 0.4 b	7.7 ± 0.3 b	358.9 ± 4.1 b
		21	17.1 ± 0.3 b	17.1 ± 0.3 b	8.3 ± 0.2 b	351.2 ± 3.8 b
10	Male	7	33.1 ± 0.7 c	33.1 ± 0.7 c	10.5 ± 0.3 c	153.2 ± 1.3 c
		14	33.6 ± 1.2 c	33.6 ± 1.2 c	10.6 ± 0.2 c	157.1 ± 2.0 c
		21	33.8 ± 1.1 c	33.8 ± 1.1 c	10.8 ± 0.2 c	154.2 ± 3.2 c
	Female	7	37.7 ± 0.3 d	37.7 ± 0.3 d	12.2 ± 0.3 d	176.6 ± 3.7 d
		14	37.3 ± 0.3 d	37.3 ± 0.3 d	12.6 ± 0.2 d	179.1 ± 3.1 d
		21	37.0 ± 1.1 d	37.0 ± 1.1 d	12.3 ± 0.1 d	179.2 ± 5.4 d
12	Male	7	51.3 ± 0.8 e	51.3 ± 0.8 e	15.2 ± 0.4 e	109.7 ± 3.9 e
		14	51.9 ± 1.4 e	51.9 ± 1.4 e	15.0 ± 0.2 e	107.8 ± 4.5 e
		21	51.2 ± 1.4 e	51.2 ± 1.4 e	15.1 ± 0.1 e	107.7 ± 4.3 e
	Female	7	57.2 ± 0.5 f	57.2 ± 0.5 f	17.6 ± 0.3 f	139.5 ± 6.5 f
		14	57.5 ± 1.1 f	57.5 ± 1.1 f	17.7 ± 0.2 f	131.9 ± 5.6 f
		21	58.2 ± 1.4 f	58.2 ± 1.4 f	17.6 ± 0.4 f	133.4 ± 7.6 f
14	Male	7	126.9 ± 1.2 g	123.9 ± 1.7 g	22.1 ± 0.3 g	13.9 ± 0.3 g
		14	126.2 ± 1.5 g	122.8 ± 2.5 g	21.8 ± 0.4 g	13.6 ± 0.4 g
		21	125.9 ± 1.3 g	123.4 ± 0.5 g	21.9 ± 0.2 g	13.9 ± 0.4 g
	Female	7	130.2 ± 1.1 h	125.9 ± 0.7 g	23.5 ± 0.1 h	15.9 ± 0.2 h
		14	132.7 ± 1.7 h	128.8 ± 1.2 g	23.9 ± 0.2 h	15.2 ± 0.1 h

		21	133.0 ± 1.2 h	125.6 ± 1.5 g	23.3 ± 0.4 h	15.6 ± 0.4 h
14.5	Male	7	150.9 ± 1.7 i	120.6 ± 1.2 g	25.1 ± 0.2 i	10.7 ± 0.3 i
		14	149.6 ± 1.4 i	120.1 ± 1.4 g	25.8 ± 0.3 i	10.5 ± 0.4 i
		21	151.7 ± 1.3 i	119.9 ± 0.9 g	25.2 ± 0.3 i	10.7 ± 0.3 i
	Female	7	157.4 ± 1.5 j	121.1 ± 1.1 g	27.4 ± 0.2 j	12.0 ± 0.2 j
		14	157.2 ± 1.3 j	122.7 ± 1.2 g	27.5 ± 0.3 j	12.1 ± 0.2 j
		21	157.6 ± 1.6 j	121.9 ± 0.9 g	27.6 ± 0.1 j	12.2 ± 0.3 j
15	Male	7	170.7 ± 1.7 k	120.5 ± 0.6 g	30.3 ± 0.2 k	7.2 ± 0.1 k
		14	171.5 ± 1.2 k	121.9 ± 0.5 g	30.2 ± 0.3 k	7.2 ± 0.1 k
		21	173.5 ± 1.3 k	119.5 ± 0.6 g	30.4 ± 0.2 k	7.3 ± 0.2 k
	Female	7	186.9 ± 1.7 l	121.6 ± 1.3 g	32.6 ± 0.3 l	8.6 ± 0.2 l
		14	185.5 ± 1.1 l	122.1 ± 1.2 g	32.8 ± 0.2 l	8.5 ± 0.2 l
		21	184.9 ± 1.4 l	129.9 ± 0.6 g	32.5 ± 0.4 l	8.4 ± 0.3 l

[1] Days after eclosion.

[2] TAEC, total amount of electric current released from a captured fly; AECD, total amount of electric current until fly death.

We used 20 insects for each sex and age. Means ± standard deviation were calculated from five experimental replicates. Different letters (a–l) within a column indicate significant differences ($P < 0.05$) according to Tukey’s test.
